# Boosting Li/Na storage performance of graphite by defect engineering†

**DOI:** 10.1039/d1ra03102c

**Published:** 2021-06-24

**Authors:** Mingyang Ou, Shixiong Sun, Yi Liu, Yue Xu, Chang Chen, Pei Hu, Chun Fang, Qing Li, Jiantao Han

**Affiliations:** State Key Laboratory of Material Processing and Die & Mould Technology, School of Materials Science and Engineering, Huazhong University of Science and Technology Wuhan 430074 P. R. China; School of Science, Hubei University of Technology Wuhan 430068 P. R. China hupei@hbut.edu.cn; State Environmental Protection Key Laboratory of Soil Health and Green Remediation Wuhan 430070 P. R. China; College of Resource and Environment, Huazhong Agricultural University Wuhan 430070 P. R. China changchen@mail.hzau.edu.cn

## Abstract

Regulating material properties by accurately designing its structure has always been a research hotspot. In this study, by a simple and eco-friendly mechanical ball milling, we could successfully engineer the defect degree of the graphite. Moreover, according to the accurate deconstruction of the structure by atomic pair distribution function analysis (PDF) and X-ray absorption near-edge structure analysis (XANES), those structural defects of the ball-milled graphite (BMG) mainly exist as carbon atom vacancies within the graphene structure, which are beneficial to enhance the lithium and sodium storage performance of BMG. Therefore, BMG-30 h exhibits superior lithium and sodium storage performance.

## Abstract 2

The structural defects of ball-milled graphite (BMG) mainly exist as carbon atom vacancies within the graphene structure, which are proven to be the main source of lithium/sodium storage performance promotion of BMGs.

## Graphical abstract

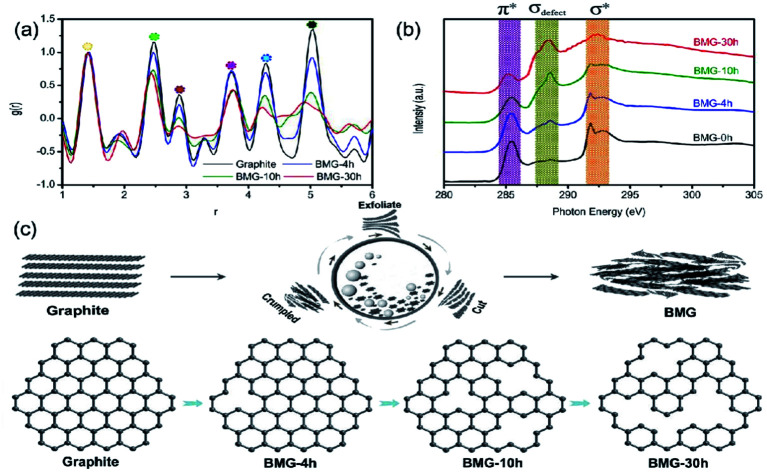

## 1. Introduction

The aggravation of environmental crisis and concerns over the limited resources of fossil fuels boost the interest in harvesting energy from renewable energy sources.^1–3^ Unfortunately, most of these renewable energy sources are intermittent; the need to effectively utilize these unsteady energies has spurred the demand of energy storage devices.^4–7^ Until now, lithium-ion batteries (LIBs), the current state-of-the-art devices in energy storage technology, are dominant in the field of portable electronic devices and electric vehicles.^8–10^ Nevertheless, when scaling up the application to the state grid systems, lithium is too rare to be deployed. Therefore, the attempt to use inexpensive and earth-abundant metal elements, such as sodium, magnesium, and aluminum, to replace lithium have aroused tremendous interest in the last few years.^11–13^ Among those batteries, sodium-ion batteries (NIBs) have drawn the most attention owing to the abundance and widely distributed resource of sodium and the similar rocking chair storage mechanism of sodium to that of lithium.^13,14^ Therefore, in recent years, NIBs have undergone a renaissance with numerous new materials and approaches quickly springing back up. At the cathode side, great progress has been made and Na_3_V_2_(PO_4_)_3_, a layered sodium metal oxide, olivine-type sodium metal phosphates, and Prussian blue have been investigated.^1,13,15^ As for the anode materials, although alloy type and conversion type materials showed high initial capacity because of the large volume change and sluggish kinetics those materials suffered from poor cyclability.^16,17^ Besides the aforementioned materials, carbon materials proposed as anode materials for SIBs can be applied in large-scale energy storage devices particularly when graphite is reported as an SIB anode.

Graphite is the dominant anode material in commercial LIBs and is regarded as the best anode material for large energy storage devices due to its abundance, low cost, electrical conductivity, chemical stability, and high reversible capacity.^14,18^ However, for a long time, only less than 35 mA h g^−1^ sodium storage capacity and 372 mA h g^−1^ lithium storage capacity severely hinder the commercial development of LIBs and SIBs.^19,20^ How to boost the lithium/sodium storage performance of graphite has become the direction of researchers' efforts.

The unfavorable mismatching of the graphite layer distance and the Na-ion diameter are generally considered to be a main reason for the low sodium storage capacity in graphite by forming a kind of low Na content graphite intercalation compound, namely NaC64.^21–26^ Encouragingly, in recent studies, researchers successfully increased the sodium storage capacity in graphite by expanding the layer space.^15,27^ Adelhelm *et al.* used a solvent-Na-ion co-intercalation into the graphite method to expand the graphite interlayer space, which can achieve a capacity of 130 mA h g^−1^ at the current density of 20 mA g^−1^ in a diglyme-based electrolyte. Furthermore, Kang *et al.* by regulating the solvent molecules of ether electrolyte obtained a capacity of 150 mA h g^−1^, which is the highest sodium storage capacity of graphite delivered in ether electrolyte. Intriguingly, Wang *et al.* used another method to expand the graphite interlayer distance by reducing the oxidized graphite. They expanded the graphite interlayer distance up to 4.3 nm and achieved a capacity of 280 mA h g^−1^ at the current density of 20 mA g^−1^. These results are encouraging; however, it is worthy to note that there are still some drawbacks among both the methods.^28–34^ For the Na-solvent co-intercalation approach, (the inserted solvents caused a high-level of volume change) not only the high-level volume caused by the solvent co-intercalation but also the relatively low capacity (below 150 mA h g^−1^) inherently limit its practical applications. As for the second method, the existing oxygen functional groups on the surface of expanded graphite layers can obstruct the transportation of Na^+^ ions and demonstrate a poor rate performance. For a current density of up to 100 mA g^−1^, the capacity drastically declined to 136 mA h g^−1^. Therefore, it is challenging but desirable to find a suitable method to improve the sodium storage capacity in graphite without sacrificing the rate performance. For mental oxide materials, the intercalation pseudocapacitive charge storage mechanism is a promising method to achieve high capacity, superior rate performance, and long life cycle stability, which have been demonstrated by Dunn *et al.*^35^ Inspired by this, it is very probable to achieve high capacity and superior rate performance by introducing an intercalation pseudocapacitive charge storage mechanism in graphite.

In this study, we used a simple and eco-friendly ball milling method to treat graphite, which yielded a series of ball-milled graphites (BMGs) with structural defects. Accurately reconstructing the structure of BMGs by PDF and NEXAS, we found that the structural defects mainly come from carbon atomic vacancies within the graphene structures, which is expected to benefit lithium/sodium storage. Therefore, BMGs exhibit superior lithium/sodium storage performance. Also, we also demonstrate that the superior sodium storage performance of BMGs come from the contribution of intercalation pseudocapacitance.

## 2. Experimental

### 2.1 Material synthesis

Commercial graphite and zirconium balls were put into a nylon ball mill jar under an argon atmosphere with a weight ratio of 1 : 40. Then, the commercial graphite was milled at the rate of 1400 rpm for 0 h, 4 h, 10 h, and 30 h. The corresponding samples are respectively labelled as BMG-0 h, BMG-4 h, BMG-10 h, and BMG-30 h.

### 2.2 Materials characterization

Desk-top high speed vibrating ball mill (MSK-SFM-3, MTI, China) was used to synthesize the samples. Empyrean Nano, the Netherlands, with Cu-Kα radiation was used to collect XRD patterns at a scan rate of 12° min^−1^, with Ag Kα radiation to get pair distribution function (PDF) patterns. The X-ray absorption near edge structure (XANES) was conducted at BL12B beamline in the National Synchrotron Radiation Laboratory (BSRL) in Hefei. Raman spectra were obtained by LabRAM HR800 using an Ar ion laser with a wavelength of 532 nm. The SEM images were recorded by VEGA 3 SBH (TESCAN, Czech Republic). TEM images were measured by Tecnai G2 F30 (FEI, USA).

### 2.3 Electrochemical measurements

The electrode was prepared by mixing BMGs, Super-P, and polymer binder (CMC : SBR = 1 : 1) at the ratio of 8 : 1 : 1 uniformly into a slurry, and coated it on the copper foil with the mass loading of BMGs about 3 mg cm^−2^. The BMG electrode was assembled as CR2032 coin-type cells in a glove box filled with Ar atmosphere, using a Li/Na metal as the counter electrode, glass fiber membrane as the separator, and 1 M LiPF_6_/NaPF_6_ in EC and DEC (1 : 1, v/v) as the electrolyte. Galvanostatic charge and discharge (GCD) tests were performed on the Land battery test system (LAND 2001 CT, China). The cyclic voltammetry (CV) tests were carried out on an electrochemical workstation (CHI760E, China).

## 3. Results and discussion

**Fig. 1:**
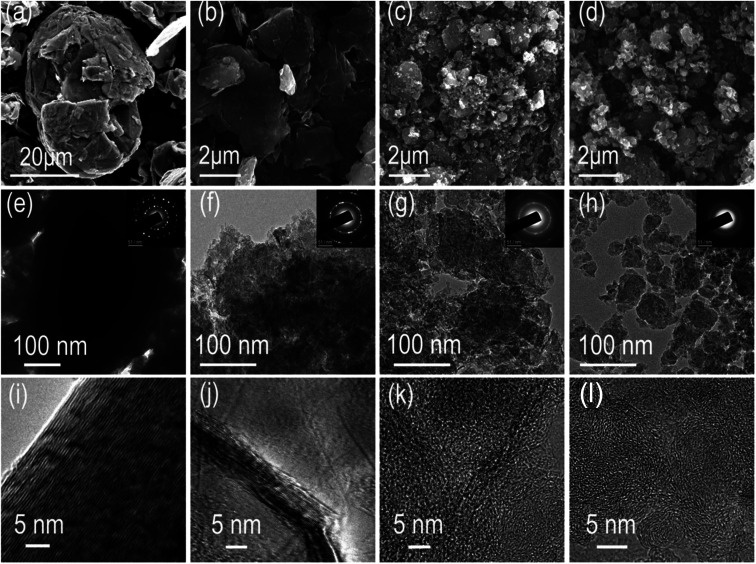
The morphology of the BMGs. (a–d) SEM images of BMG-0 h, BMG-4 h, BMG-10 h, and BMG-30 h. (e–h) TEM and the corresponding SAED images of BMG-0 h, BMG-4 h, BMG-10 h, and BMG-30 h. (i–l) HRTEM images of BMG-0 h, BMG-4 h, BMG-10 h, and BMG-30 h.

Mechanical ball milling is chosen for manufacturing defects in graphite due to its facile control and eco-friendly nature. A series of ball-milled graphite (BMG) was prepared by varying the mechanical ball milling time, denoted as BMG-0 h, BMG-4 h, BMG-10 h, and BMG-30 h. The morphology of BMGs was characterized by scanning electron microscopy (SEM). As shown in Fig. 1a–d, the morphology and particle sizes of BGMs show significant changes as the milling time increased; BMG-0 h, as the starting material, consisted of flakes with the particle size ranging from 20 to 50 μm. After 4 h of mechanical ball milling, the main morphology of BMG-4 h shown in Fig. 1b is a multilayer graphene sheet with a particle size of 2–5 μm, which were exfoliated from the flakes of graphite. As the ball milling time increased to 10 h, as shown in Fig. 1c, the major morphology of BMG-10 h was particles with a particle size of about 200 nm, due to the cutting and crumpling by mechanical ball milling. The morphology of BMG-30 h demonstrated in Fig. 1d is similar to that of BMG-10 h but with smaller particle sizes. The structural changes of BMGs were further detected *via* transmission electron microscopy (TEM). The TEM images of BMGs, as shown in Fig. 1e–h, depict similar morphology and particle size evolution trends to those of the SEM images. However, the high-resolution TEM images (Fig. 1i–l) demonstrate that in addition to the morphology and particle size changes, the crystal structure of the material had also undergone a significant change. As shown in Fig. 1i, the well-defined layers of the paralleled lattice fringes for BMG-0 h indicate its intact crystal structure. As for BMG-4 h, although the lattices fringes are similar to those of BMG-0 h, the structure of well-defined layers was broken and destroyed; the disrupted crystal defects are easily visible in Fig. 1j. As ball milling conditions increase to 10 h, in Fig. 1k, long-range order well-defined layers paralleled lattices fringes are disappeared which are replaced by short-range order draped layers lattices fringes shown with a bend layer with many breakages and crumples. Furthermore, when the milling time increased to 30 h, as shown in Fig. 1l, the graphite crystal structure disrupted sufficiently, forming a totally disordered pattern with a micro-porous microtexture. From the HRTEM images of BMG samples, we can find that with an increase in the milling time, the crystal structure of graphite disrupted more and more. This trend is also verified by SAED characterization. This means that by simply controlling the mechanical ball milling time, we successfully introduced different degrees of crystal structural defects into graphite.

**Fig. 2:**
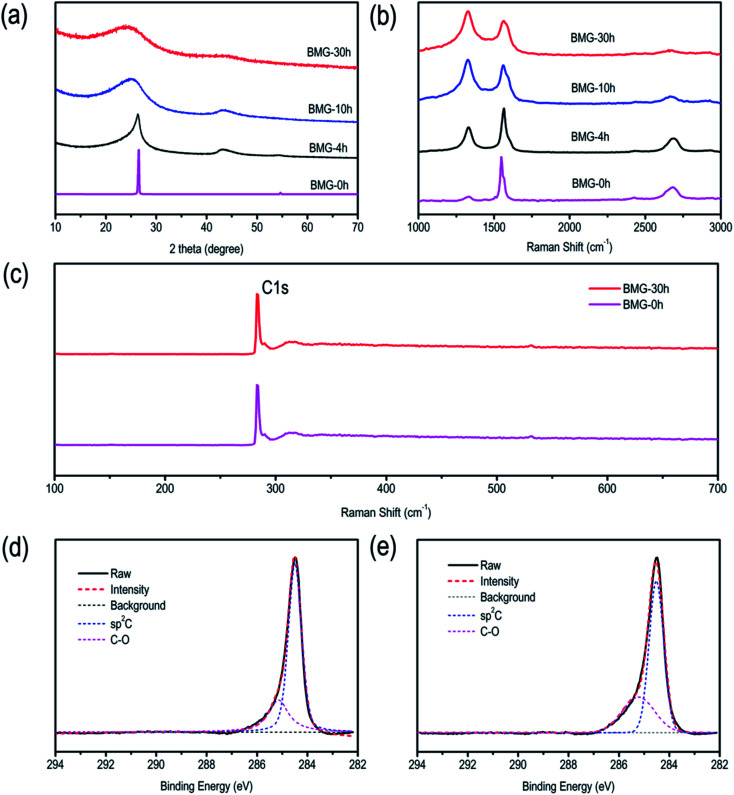
(a) XRD patterns of BMGs. (b) Raman spectra of BMGs. (c) XPS survey spectra. (d) High-resolution C 1s spectra of BMG-0 h. (e) High-resolution C 1s spectra of BMG-30 h.

The crystal structural evolution trend of BMGs is also characterized *via* powder X-ray diffraction, and the results are shown in Fig. 2a. BMG-0 h exhibits a sharp peak at 2*θ* = ∼26.6°, which corresponded to the diffraction of the (002) plane with an interlayer distance of 0.34 nm. Moreover, we can find that with the increase in the ball mill time, the diffraction peak of the (002) plane showed a blue-shift by a small angle, indicating that the interlayer distance of BMGs was expanded by the sheer force of mechanical ball milling. In addition, it should be noted that the full width at half maximum (FWFM) for the diffraction of the (002) plane became wider, indicating that the particle of graphite was cut by the sheer force of mechanical ball milling, which is consistent with the results of SEM and TEM.

Raman spectra of BMGs are shown in Fig. 2b. We can find that the spectra of graphite show a typical G band located at 1601 cm^−1^ and a weak D band at 1372 cm^−1^, which are attributed to the vibrations of sp^2^ bonded carbon atoms and the disordered carbon atoms with a defect in a two-dimensional hexagonal lattice, respectively.^7,36^ Nevertheless, with an increase in the ball mill time, the ratio of *I*_D_/*I*_G_ grew, which demonstrated the more disorderly structure of BMGs.

**Fig. 3:**
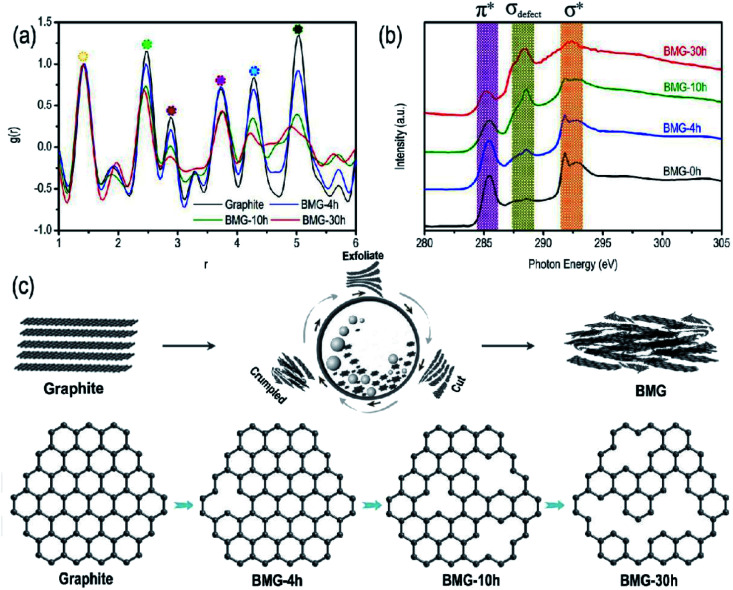
(a) PDF patterns of BMGs. (b) C 1s NEXAS patterns of BMGs. (c) Schematic of the structural transformation of BGMs.

The atomic pair distribution function analysis (PDF) is often used to accurately resolve the crystal structure change at the atomic scale. In this study, PDF was adopted to intuitively quantify the crystal structural changes of BMGs. The PDF results of BMGs are shown in Fig. 3a in the range of 6-atom shells. The peaks in Fig. 3a in turn correspond to each atomic shell of graphene around the central atom, which is schematically shown in Fig. S1† with the peak area corresponding to the number of shell atoms. Compared with the PDF curve of graphite, we can find that as the milling time increased, the peak locations remained almost unchanged but the peak area of BMGs decreased significantly, indicating that the graphene structure of BMGs did not change and the structural defects of BMGs mainly comes from the carbon atomic vacancies within the graphene structure, which are introduced into the crystal structure by ball milling. The same conclusion is also derived from the result of the X-ray absorption near edge structure analysis (XANES). As displayed in Fig. 3b, with the increase in the milling time, the peaks of π* + σ*, which are due to the presence of sp^2^ bonding expected for the graphene structure decreased obviously, but the peaks of σ_defect_, which are due to the presence of defect bonding in the graphene structure increased significantly, indicating that ball milling is an effective and controllable method to introduce structural defects into the graphite structure. The structural evolution process of BMGs with ball milling time is schematically shown in Fig. 3c; by exfoliating, cutting, and crumpling, mechanical ball milling can introduce numerous carbon atom vacancies among the graphene structure of BMGs. According to the previous literature, the graphene or local graphene-like structural carbon materials with numerous carbon atom vacancies have been expected to be the most suitable electrode material for lithium/sodium storage.

**Fig. 4:**
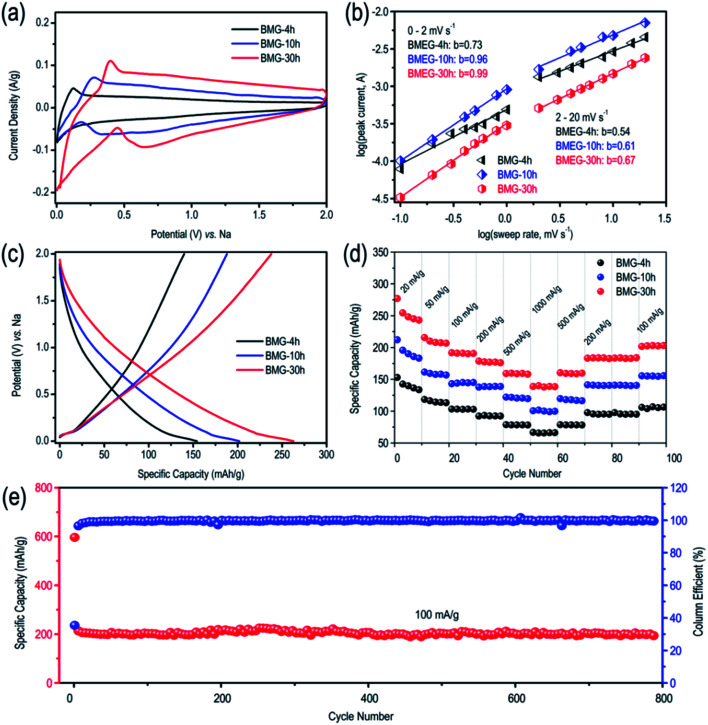
(a) CV curves of BMGs at the scan rate of 1 mV s^−1^. (b) *b*-value of the cathodic currents peak of BMGs. (c) GCD curves of BMGs at the current density of 20 mA g^−1^. (d) Specific capacities of BMGs at different current densities. (e) Cycling stability test of BMG-30 h at the current density of 100 mA g^−1^.

The lithium storage performance of BMG is shown in Fig. S2.† At the current density of 100 mA g^−1^, BMG-0 h, BMG-4 h, BMG-10 h, and BMG-30 h exhibit the capacity of 370 mA h g^−1^, 580 mA h g^−1^, 935 mA h g^−1^ and 1130 mA h g^−1^, respectively, demonstrating that the carbon atom vacancies distributed in the graphene structure greatly boost the lithium storage performance of BMGs. Moreover, the lithium storage capacity of BMGs increases with the increase in the concentration of the carbon atom vacancies among the graphene structure. As for the sodium storage performance of BMGs, as displayed in Fig. 4a, the pair of obvious reversible peaks appearing in the cyclic voltammetry (CV) curves of BMG-4 h, BMG-10 h, and BMG-30 h are representative of an ideal efficient sodium de-intercalation. In addition, as shown in Fig. 4c, BMG-4 h, BMG-10 h, and BMG-30 h display a sodium storage capacity of 156 mA h g^−1^, 202 mA h g^−1^, and 271 mA h g^−1^ at the current density of 20 mA g^−1^, respectively, which are significantly higher than the theoretical sodium storage capacity of graphite. To better understand the superior sodium storage performance of BMGs, the electrochemical kinetics of BMG electrodes was evaluated based on the cycle voltammetry (CV) analysis. Fig. S3† displays the CV curves of BMG-4 h, BMG-10 h, and BMG-30 h at various sweep rates. The relationship between the measured current (*i*) and the sweep rate (*v*) is as follows:*i* = *av*^*b*^,where *a* and *b* are the adjacent values. The *b*-value is determined by the slope of the log(*v*)–log(*i*) plots and related to the type of the sodium reaction process. When the *b*-value is 0.5, it indicates a total diffusion-controlled reaction process, whereas the *b*-value of 1 represents a surface-controlled capacitive process.^28^Fig. 4b presents the log(*v*)–log(*i*) plots of BMG electrodes for cathodic peaks. For sweep rates ranging from 0.1–20 mV s^−1^, the *b*-value for BMG-30 h is 0.99, indicating that the kinetics of BMG-30 h exhibits surface-controlled capacitive characteristics. Compared with the *b*-values of BMG-4 h and BMG-10 h, which were 0.73 and 0.96, respectively, we can easily conclude that longer the milling time, the kinetics of BMGs are more surface controlled. These results explained why BMG-30 h possesses a superior rate performance and cycle stability, which are displayed in Fig. 4d and e. Fig. 4b also exhibits a change in the slope of the log(*v*)–log(*i*) plots at 2 mV s^−1^. The *b*-value of BMG-30 h decreased to 0.67 when sweeping rates were >2 mV s^−1^; moreover, the *b*-value of BMG-10 h and BMG-30 h decreased to 0.61 and 0.54, respectively. Similar observations were reported by previous studies; the limitation to the rated capacity should be attributed to an increase in the diffusion constraints under an ultra-fast scan rate. The higher *b*-value of BMG-30 h compared to BMG-4 h and BMG-10 h further demonstrates that the higher structural defect significantly increases the adsorption capacity, which is the main sodium storage capacity of BMGs.

**Fig. 5:**
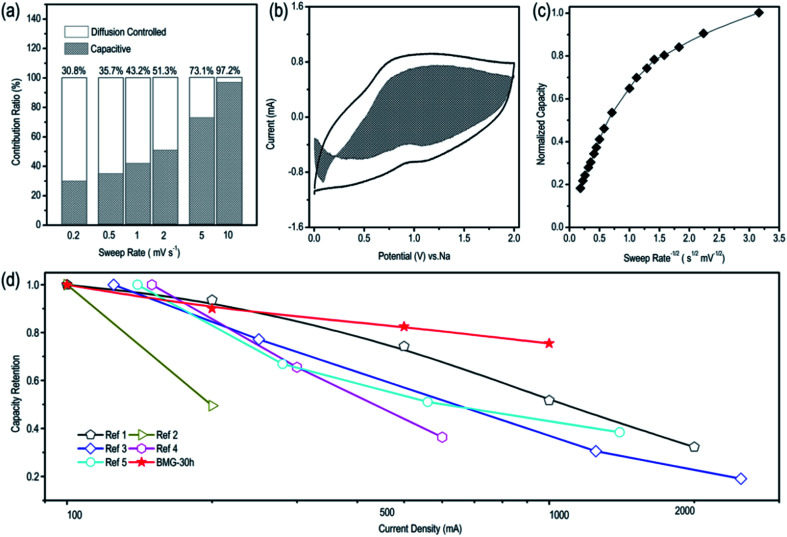
(a) Contribution ratios of the adsorption capacity at different scan rates of BMG-30 h. (b) Capacitive contribution of BMG-30 h at the scan rate of 5 mV s^−1^. (c) The plot of normalized capacity *versus v*^−1/2^ for BMG-30 h. (d) Capacity retention of BMG-30 h *versus* other carbon materials.

To better understand the sodium storage behavior of BMG-30 h, originating from the reversible sodium-ion adsorption/desorption on the carbon atomic vacancies, the equation of *i* = *k*_1_*v* + *k*_2_*v*^1/2^ was adopted to calculate the contribution ratios of adsorption capacity at different scan rates, where *k*_1_*v* is the capacitive contribution and *k*_2_*v*^1/2^ is the diffusion-limited contribution.^28^ The results are displayed in Fig. 5a and the capacitive contribution curve of BMG-30 h at 5 mV s^−1^ is shown in Fig. 5b. The plot of capacity *versus v*^−1/2^ of BMG-30 h is shown in Fig. 5c. As the sweep rate in the range of 0.1–1 mV s^−1^, the capacity varied insignificantly, indicating that the capacitive contributions are independent of the sweep rate. As for the sweep rate from 1 to 20 mV s^−1^, the capacity decreased linearly with *v*^−1/2^, which indicates that at high sweep rates, the sodium-ion storage is mainly from capacitive contributions. The comparison of the rate capability of BMG-30 h with that of other carbon materials is shown in Fig. 5d; the high rate capability of BMG-30 h implies that the capacitive dominated storage mechanism permits exceptionally rapid sodium ion transport.

## 4. Conclusions

In summary, in this study, mechanical ball milling was chosen to introduce defects into the graphite crystal structure. By using PDF and NEXAS methods to accurately analyze the defects of BMGs, we found that the structural defects mainly come from the carbon atomic vacancies within the graphene structures, which have been expected to benefit lithium/sodium storage. As a result, BMG-30 h, with the largest carbon atom vacancies, exhibits excellent lithium and sodium storage performance. Thus, this study not only presents a highly attractive practical anode for LIBs and NIBs, but also provides a new insight into designing carbonaceous materials with superior sodium storage performance.

## Conflicts of interest

There are no conflicts to declare.

## Supplementary Material

RA-011-D1RA03102C-s001
