# Supplementary material for: Boosting Li/Na storage performance of graphite by defect engineering
Source: RSC Adv. 2021 Jun 24;11(36):22297–304. doi: 10.1039/d1ra03102c (PMC9034195; doi:10.1039/d1ra03102c)
Supplement: RA-011-D1RA03102C-s001 [file RA-011-D1RA03102C-s001.pdf]

Electronic Supplementary Material (ESI) for RSC Advances.
This journal is © The Royal Society of Chemistry 2021

Electronic Supplementary Information

Boosting Li/Na Storage Performance of Graphite by Defect Engineering

Mingyang Ou[a]‡, Shixiong Sun[a]‡, Yi Liu[a], Yue Xu[a], Chang Chen[c, d]*, Pei Hu[a, b]*, Chun Fang[a], Qing Li[a] and Jiantao Han[a]

[a.] State Key Laboratory of Material Processing and Die &Mould Technology School of Materials Science and Engineering, Huazhong University of Science and Technology, Wuhan 430074, P. R. China

[b.] School of Science, Hubei University of Technology, Wuhan 430068, P. R. China

[c.] State Environmental Protection Key Laboratory of Soil Health and Green Remediation, Wuhan 430070, P. R. China

[d.] College of Resource and Environment, Huazhong Agricultural University, Wuhan 430070, P. R. China

Corresponding Author E-mail: hupei@hbut.edu.cn (Prof. Hu), changchen@mail.hzau.edu.cn (Dr. Chen)

‡M. Ou and S. Sun contributed equally to this work.

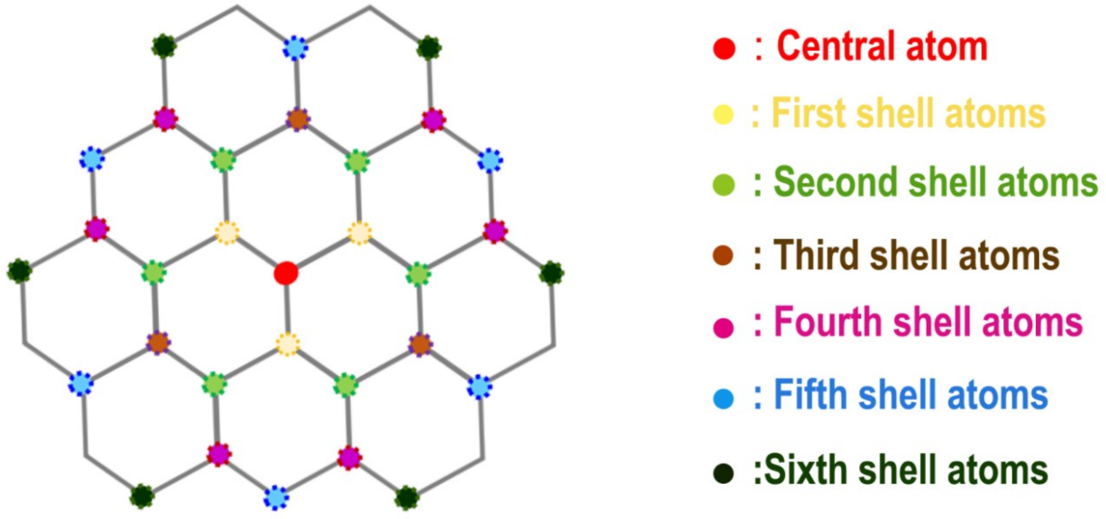

Fig. S1. Schematic illustration of 6-atom shells graphene unit.

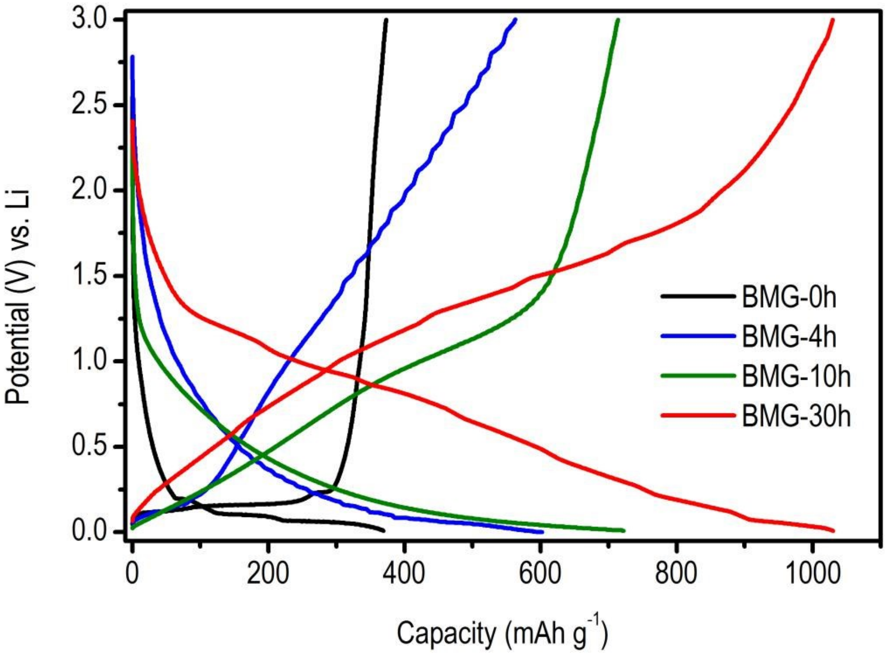

Fig. S2. GCD curves of BMGs at the current density of 20 mA g^{-1}.

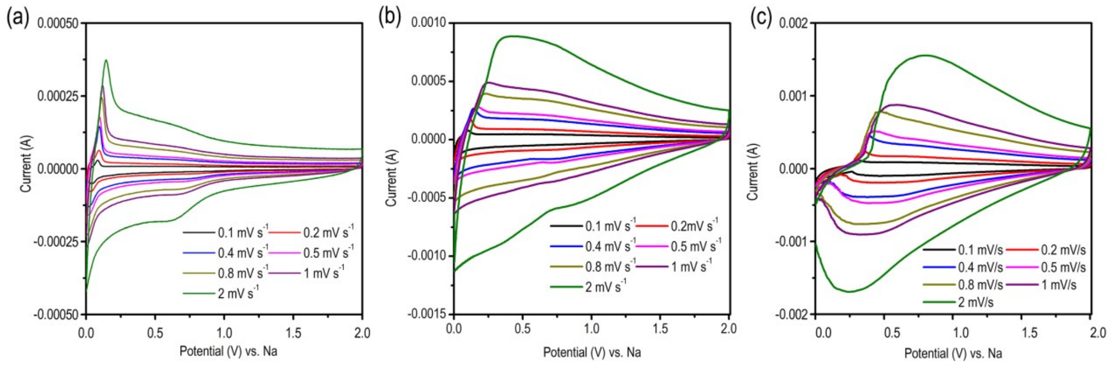

Fig. S3.CV curves of BMGs at different scan rate. (a) BMG-4h, (b) BMG-10h, (c) BMG-30h.
